# Relationship Between Computed Tomography Findings and Neutrophil-to-Lymphocyte Ratio in Mild Head Trauma Cases

**DOI:** 10.7759/cureus.79726

**Published:** 2025-02-26

**Authors:** Ezgi Akar, Dilara B Sagiri, Eylem Burcu K Özlü, Selin Tural

**Affiliations:** 1 Neurosurgery, Haydarpasa Numune Training and Research Hospital, Istanbul, TUR

**Keywords:** ct, head trauma, lymphocyte, neutrophil, nlr

## Abstract

Aim: It is known that hematological parameters increase after head trauma and have a poor prognosis. The neutrophil-to-lymphocyte ratio (NLR) is an independent prognostic factor in predicting the outcome of patients with head trauma. In our study, we investigated whether there is a correlation between computed tomography (CT) findings and NLR in cases of mild head trauma (Glasgow Coma Scale (GCS) score: 14-15).

Material and methods: We retrospectively analyzed 133 cases of head trauma with a GCS score of 14-15 admitted to the emergency department of our hospital. The cases were grouped as normal, scalp lesions, fracture (facial bone fractures, convexity, skull base), and hemorrhage (epidural hematoma, subdural hematoma, subarachnoid hemorrhage) according to CT findings. CT findings and NLR values were compared statistically.

Results: While 60.9% (81 patients) had normal CT findings, 6% (eight patients) had traumatic subarachnoid hemorrhage, 1.5% (two patients) had epidural hematoma, 1.5% (two patients) had subdural hematoma, 0.8% (one patient) had calvarial fractures, 19.5% (26 patients) had scalp lesions, and 6.8% (nine patients) had nasal fractures. There was no statistically significant relationship between NLR, GCS, and age of the patients. There was no statistically significant difference between the CT findings of the patients in terms of NLR measurements.

Conclusion: Although NLR values increased in patients with mild head trauma, no statistically significant increase was observed. No significant difference was found between NLR values when the cases were grouped according to CT findings.

## Introduction

Traumatic brain injuries (TBI) may result in psychological and socioeconomic losses as well as severe disabilities. Approximately 30-50% of TBI in childhood and adolescence are fatal [1]. Primary cerebral damage at the time of the event due to the trauma effect is inevitable. However, secondary brain damage due to oxidative stress, ischemia, brain edema, and inflammation caused by trauma can be prevented [1,2]. Although the cause and mechanism of inflammation are still unclear, it is known that neutrophils, astrocytes, microglia, and monocytes trigger the release of many proinflammatory cytokines and angiogenic factors, leading to neuronal damage. Uncontrolled release of cytokines and proinflammatory mediators disrupts the endothelial structure and results in the disruption of the blood-brain barrier, increase in interstitial fluid, and leukocyte infiltration [2,3]. Disruption of the structure of the blood-brain barrier and neuronal inflammatory response cause migration of neutrophils to the area of damage in the first hour after cerebral injury, which affects the distribution of leukocytes and neutrophils in the blood circulation [4].

White blood cell (WBC) count in the blood increases in TBI, after subarachnoid hemorrhage, and in delayed cerebral ischemia [5]. The increased neutrophil-to-lymphocyte ratio (NLR), i.e., increased neutrophil count and relatively decreased lymphocyte count, is also an independent risk factor after subarachnoid hemorrhage, in Alzheimer's disease, stroke, and many cerebral inflammatory diseases [6]. It has been found that increased NLR after TBI affects clinical course and prognosis and is a poor prognostic marker [5,6]. Some studies have shown that NLR increases in parallel with the severity of head trauma and Glasgow Coma Scale (GCS) score, but the role of NLR in treatment and prognosis still needs to be developed [7].

Since computed tomography (CT) findings and the severity of injury may not always align with the GCS score of the patient, it prompted us to evaluate the relationship between NLR and CT findings when different results (normal, fracture, hematoma, contusion) were detected upon CT in patients with mild head trauma. Our study investigates if there is a correlation between CT findings and NLR in mild head trauma cases (GCS score: 14-15).

## Materials and methods

In this retrospective study, only cases with head trauma admitted to the emergency department between 2019 and 2024 were evaluated. A consent form for the retrospective study was obtained from the Ethics Committee of Haydarpasa Numune Training and Research Hospital. Patients under 18 years of age, patients with general body trauma, patients who did not present within the first six hours after trauma, patients using anticoagulants or immunosuppressive agents, patients who were intoxicated upon presentation, patients with low platelet counts, and patients with a GCS score of <14 were excluded from the study. Patients had to be CT scan-confirmed cases of TBI. CT signs of TBI included epidural hematoma, subdural hematoma, intraparenchymal hemorrhage, brain contusion, and subarachnoid hemorrhage. All patients were evaluated and treated by the same neurosurgeons with specific training in critical care, following guidelines for the management of mild TBI.

In patients with mild head trauma (GCS score: 14-15) included in the study, age, gender, GCS score, and CT findings at presentation were recorded. According to CT findings, the cases were grouped as normal, scalp lesions, fractures (facial bone fractures, convexity, skull base), and hemorrhage (epidural hematoma, subdural hematoma, subarachnoid hemorrhage) groups. NLR ratios were recorded based on neutrophil and lymphocyte values obtained from blood samples taken in the first six hours. The correlation between CT findings and NLR was evaluated statistically.

Statistical analyses were performed using IBM SPSS Statistics for macOS, version 29.0 (IBM Corp., Armonk, US). The characteristics of patients were reported as n (%), mean±SD, and median (minimum-maximum) for categorical and continuous variables, respectively. The Mann-Whitney U test was used to compare the measurement values of two independent groups, while the Kruskal-Wallis H test was used to compare the measurement values of three or more independent groups. Spearman correlation analysis was used to compare continuous variables. The p-value was set at <0.05 for statistical significance.

## Results

**Figure 1:**
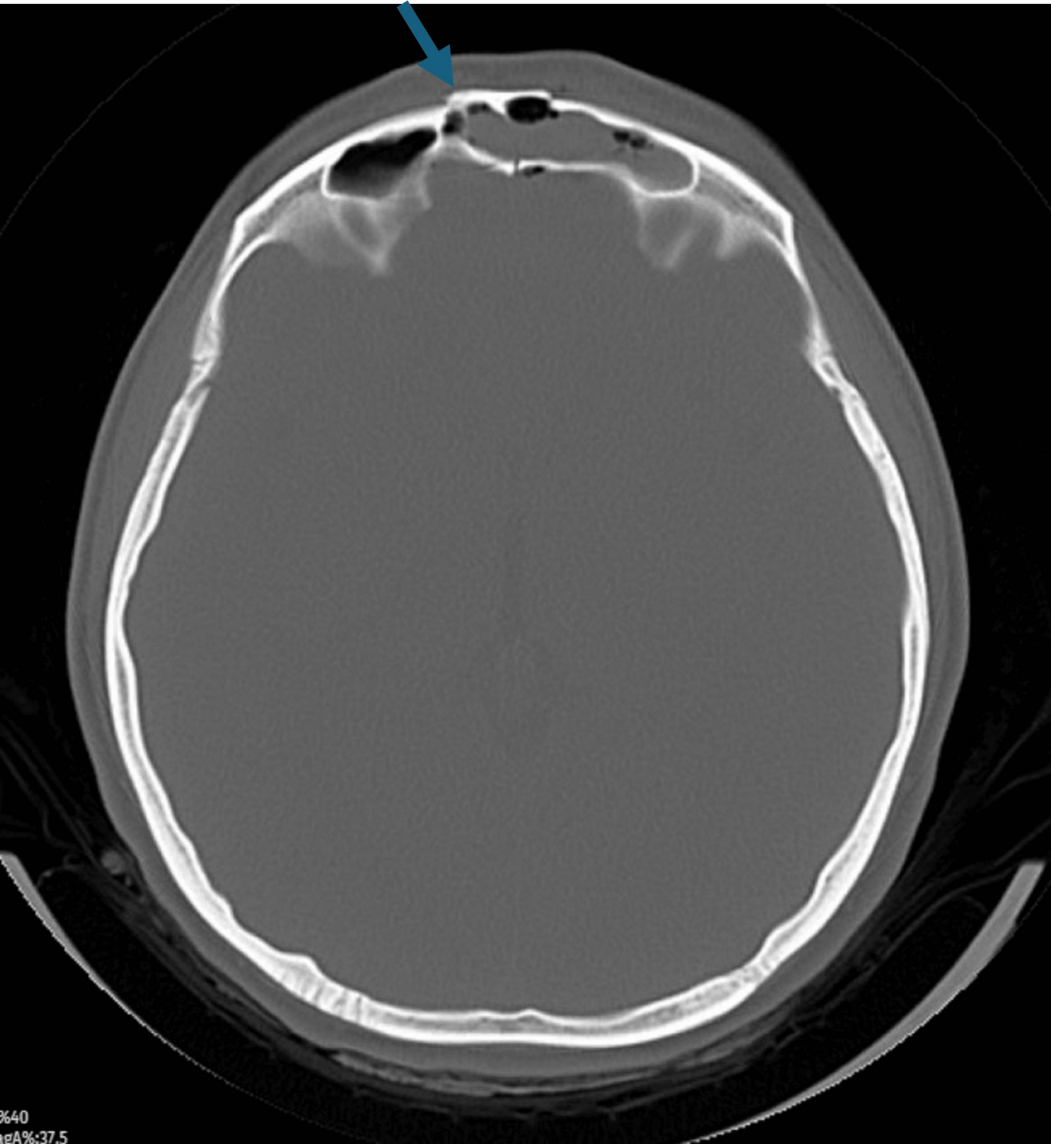
Frontal sinus anterior and posterior wall fracture.

**Figure 2:**
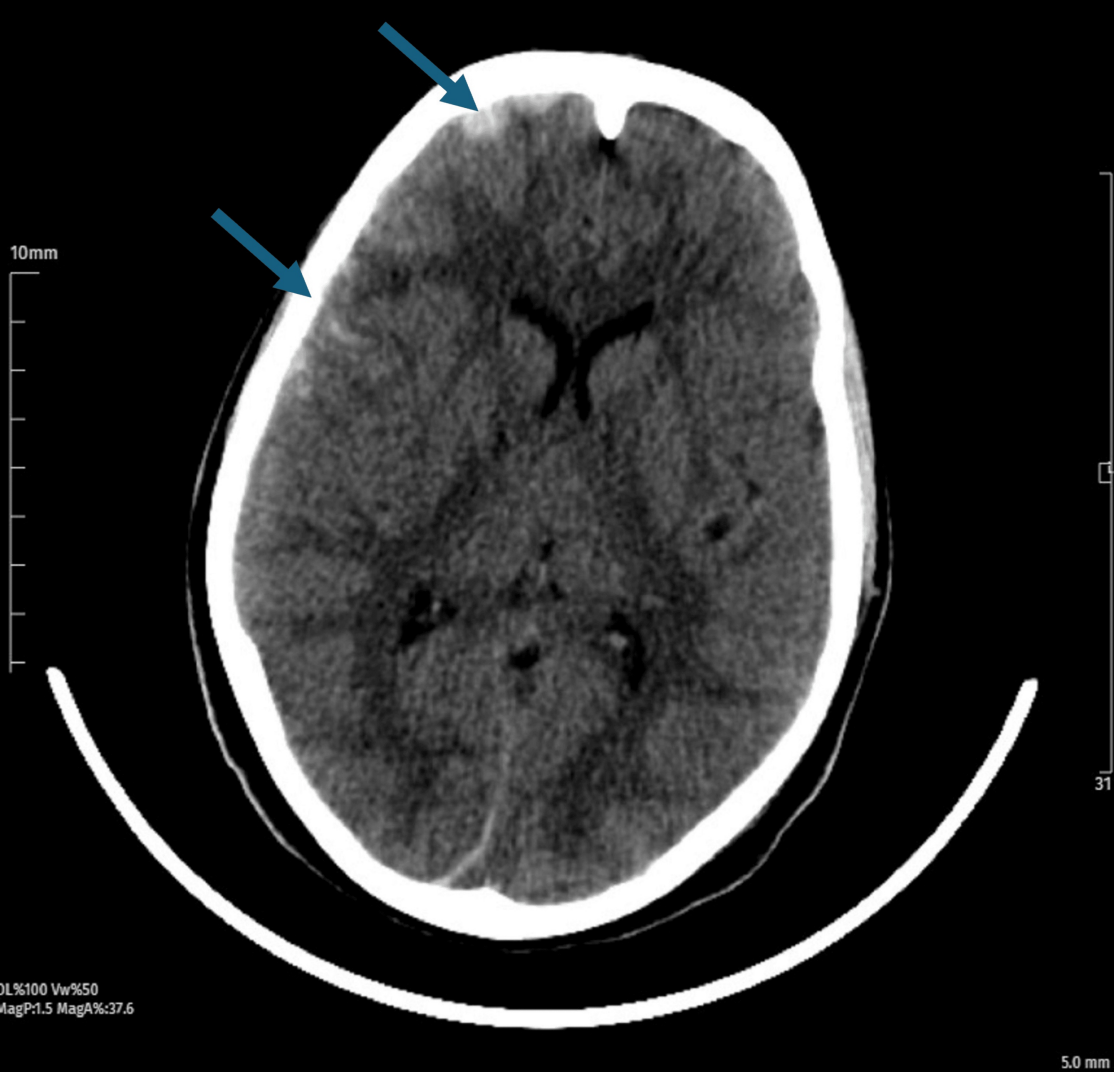
Right frontoparietal traumatic subarachnoid hemorrhage.

**Figure 3:**
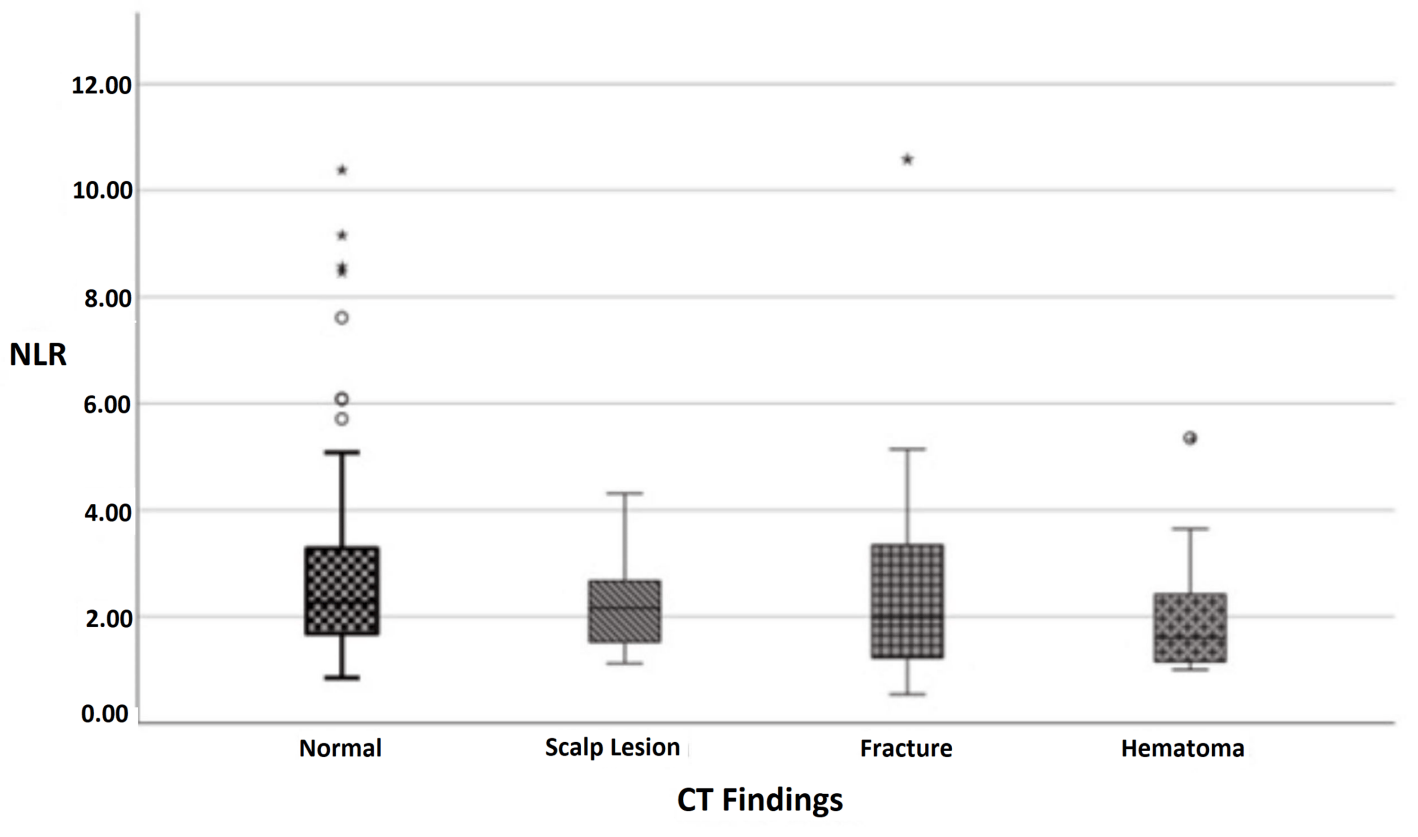
Distribution of NLR measurements according to CT findings of the patients. NLR: Neutrophil-to-lymphocyte ratio; CT: Computed tomography

A total of 133 patients, 47.4% male (63 patients) and 52.6% female (70 patients), were included in the study. The distribution of demographic and clinical findings of the patients is shown in Table 1. The ages of the patients ranged between 16-91 years and the mean and median age was 47 years. The most common clinical findings were headache in 39.1% (52 patients), scalp lesions in 27.8% (37 patients), and post-traumatic memory loss in 12% (16 patients). While 60.9% (81 patients) had normal CT findings, 6% (eight patients) had traumatic subarachnoid hemorrhage, 1.5% (two patients) had epidural hematoma, 1.5% (two patients) had subdural hematoma, 0.8% (one patient) had calvarial fractures, 19.5% (26 patients) had scalp lesions, and 6.8% (nine patients) had nasal fractures (Table 1, Figures 1-3).

**Table 1:** Distribution of NLR measurements according to CT findings of the patients NLR: Neutrophil-to-lymphocyte ratio; CT: Computed tomography; ^1^: Nasal fracture, orbital fracture, calvarial fracture, mandibular fracture, and maxillary sinus fracture; ^2^: Traumatic subarachnoid hemorrhage, epidural hematoma, and subdural hematoma

	NLR		p-value
	Mean±SD	Median (Min-Max)	
CT findings			0.288
Normal	2.83±1.95	2.27 (0.85-10.38)	
Scalp lesion	2.21±0.81	2.16 (1.12-4.31)	
Fracture^1^	2.75±2.59	1.98 (0.54-10.58)	
Hematoma^2^	2.09±1.29	1.6 (1-5.35)	

The distribution of NLR of the patients according to gender is presented in Table 2. Upon analysis, it was determined that there was no statistically significant difference between the genders of the patients in terms of NLR measurements (Table 2). The mean NLR value was 2.7±1.92 in male patients and 2.58±1.74 in female patients, which was above the normal NLR value (mean male: 1.63, female: 1.66) [8,9]. The results of the correlation analysis, the relationship between the NLR, GCS score, and age of the patients, are shown in Table 3. Upon analysis, it was determined that there was no statistically significant correlation between NLR, GCS score, and age of the patients (Table 3).

**Table 2:** NLR distribution of patients according to gender NLR: Neutrophil-to-lymphocyte ratio

	Male (n=63)	Female (n=70)	p-value
NLR			0.838
Mean±SD	2.7±1.92	2.58±1.74	
Median (Min-Max)	2.23 (0.54-10.58)	2.26 (0.81-10.38)	

**Table 3:** Distribution of demographic and clinical findings of the patients GCS: Glasgow Coma Scale; SAH: Subarachnoid hemorrhage; CT: Computed tomography; NLR: Neutrophil-to-lymphocyte ratio

Characteristics (N=133)	n (%)	Mean±SD	Median (Min-Max)
Gender			
Male	63 (47.4%)		
Female	70 (52.6%)		
Age (y)		47±24	47 (16-91)
GCS score		14.9±0.3	15 (14-15)
NLR		2.64±1.82	2.25 (0.54-10.58)
Clinical findings			
Headache	52 (39.1%)		
Scalp lesion	37 (27.8%)		
Post-traumatic amnesia	16 (12%)		
Periorbital findings	8 (6%)		
Vertigo	6 (4.5%)		
Lethargy	4 (3%)		
Seizure	4 (3%)		
Syncope	4 (3%)		
Epistaxis	1 (0.8%)		
Vomiting	1 (0.8%)		
CT findings			
Normal	81 (60.9%)		
Scalp lesion	26 (19.5%)		
Nasal fracture	9 (6.8%)		
Orbital fracture	2 (1.5%)		
Calvarial fracture	1 (0.8%)		
Mandibular fracture	1 (0.8%)		
Maxillary sinus fracture	1 (0.8%)		
Traumatic SAH	8 (6%)		
Epidural hematoma	2 (1.5%)		
Subdural hematoma	2 (1.5%)		

The distribution of NLR of the patients according to CT findings is presented in Table 4. Upon analysis, it was determined that there was no statistically significant difference between the CT findings of the patients in terms of NLR measurements (Table 4).

**Table 4:** Distribution of the relationship between NLR, GCS, and age Correlation r value between -1 and 1. p<0.05 is considered statistically significant. GCS: Glasgow Coma Scale; NLR: Neutrophil-to-lymphocyte ratio

		GCS	Age	NLR
GCS	r	1.000	-0.131	0.058
	p		0.132	0.509
Age	r	-0.131	1.000	0.131
	p	0.132		0.134
NLR	r	0.058	0.131	1.000
	p	0.509	0.134	

## Discussion

In TBI cases, increased free radicals, cytokines such as interleukin (IL)-6 and tumor necrosis factor (TNF), as well as increased WBC counts - especially neutrophil counts - are detected due to enhanced inflammatory response [6,7]. The inflammatory response that develops after the initial injury leads to secondary brain damage. Since an increase in the number of neutrophils is an indicator of acute inflammation, the main leukocyte type that increases after TBI is neutrophil. Therefore, an increase in NLR is expected due to the rising neutrophil count.

The increased NLR ratio, which indicates a heightened inflammatory response in TBI, has gained attention as a prognostic marker [8,9]. NLR, which provides important information about complex inflammatory activity in the vascular bed, is an established marker of systemic inflammation and is easily calculated. Studies have reported that a low GCS score is associated with elevated NLR ratios [10]. In other words, high neutrophil counts and relatively decreased lymphocyte counts are associated with poor prognosis after head trauma [10,11]. One study found that the neutrophil count was associated with early neurologic status and deterioration while the lymphocyte count exacerbated cerebral inflammation and brain injury [10-12].

In healthy adults, NLR values are accepted as 1.63 in men and 1.66 in women [8,9]. In our study, NLR values measured within the first six hours post-trauma were found to be higher than normal, despite a GCS score of 14-15. This indicates that NLR may be affected in mild head trauma cases, similar to patients with low GCS scores. NLR has been recognized as an independent risk factor in the prognosis of head trauma cases. Increased NLR at the time of hospital admission has been identified as a poor predictor of six-month and one-year prognosis [11]. Numerous studies have compared NLR values with GCS findings, demonstrating an inverse relationship between them [5,9,11,12]. An increase in NLR is observed in patients with lower GCS values; therefore, a poor prognosis is expected [5,9,11-13].

As is well-known, CT findings and GCS values at the presentation of trauma patients are additional markers used to predict post-traumatic prognosis [5,6,10]. In patients with mild head trauma, the prognosis expectation is generally good, but CT findings can vary. Some patients may present with normal CT findings, while others may show intraparenchymal hemorrhage that does not align with their GCS scores. This made us consider that NLR rates might vary in patients with mild head trauma. In similar studies focused on mild head trauma, it was observed that increased NLR values may correlate with positive CT findings [8,12]. According to one study, CT scans could be warranted in patients with good consciousness (GCS score ≥14) based on increased NLR rates (7). However, other studies have indicated that poor CT findings (such as severe hemorrhage appearances) do not always correlate with the patient's GCS score [8,13-15]. Although studies have shown that increased NLR correlates with positive CT findings, it remains unclear whether there are differences between the findings detected on CT.

Reviewing the literature, it appears that a decrease in GCS score and an increase in NLR are correlated, suggesting that admission NLR should not be very different from normal limits in mild trauma cases with GCS scores of 14-15 [10,13]. In our study, although a slight increase in NLR values was observed at 2.7±1.92 in male patients and 2.58±1.74 in female patients, no statistically significant difference was found. Therefore, we expected normal or near-normal NLR and fewer positive CT findings in mild head trauma cases. As a matter of fact, in our study, consistent with the literature, no difference in NLR was detected between patients with GCS scores of 14-15, which we believe is related to the severity of the trauma. As our study was performed among mild trauma cases, we believe that there is no correlation between CT findings and NLR.

The limitations of our study include the lack of a homogeneous patient group due to an insufficient number of patients. The number of cases involving epidural and subdural hematomas was quite low, and there were no cases of intracerebral hemorrhage. Our findings can be further supported by a multicenter study featuring a more homogeneous distribution, a larger number of cases, and more comprehensive prognostic data.

## Conclusions

We investigated the relationship between NLR and CT findings independently of the GCS score. As per our results, no significant relationship was found between CT findings and NLR in mild head trauma cases. In other words, it does not seem possible to predict CT findings in patients with mild head trauma according to their admission NLR. However, it would be appropriate to support this study with larger patient groups.
